# Enhanced CD25^+^Foxp3^+^ regulatory T cell development by amodiaquine through activation of nuclear receptor 4A

**DOI:** 10.1038/s41598-017-17073-y

**Published:** 2017-12-05

**Authors:** Hee Yeon Won, Ji Hyun Shin, Sera Oh, Hana Jeong, Eun Sook Hwang

**Affiliations:** College of Pharmacy and Graduate School of Pharmaceutical Sciences, Ewha Womans University, Seoul, 03760 Korea

## Abstract

CD4^+^ T cells play key roles in the regulation of immune responses against pathogenic infectious antigens via development into effector T helper and induced regulatory T (iTreg) cells. Particularly, CD4^+^CD25^+^Foxp3^+^ iTreg cells are crucial for maintaining immune homeostasis and controlling inflammatory diseases. Anti-inflammatory drugs that enhance iTreg cell generation would be effective at preventing and treating inflammatory and autoimmune diseases. In this study, we examined whether anti-malarial and anti-arthritic amodiaquine (AQ) could affect iTreg cell development. Despite the anti-proliferative activity of AQ, AQ only moderately decreased iTreg cell proliferation but substantially increased IL-2 production by iTreg cells. Furthermore, AQ dose-dependently increased iTreg cell development and significantly upregulated iTreg cell markers including CD25. Interestingly, CD25 expression was decreased at later stages of iTreg cell development but was sustained in the presence of AQ, which was independent of IL-2 signaling pathway. AQ directly increased CD25 gene transcription by enhancing the DNA-binding and transcriptional activity of nuclear receptor 4 A. Most importantly, *in vivo* administration of AQ attenuated inflammatory colitis, resulted in the increased iTreg cells and decreased inflammatory cytokines. The ability of anti-malarial AQ to potentiate iTreg cell development makes it a promising drug for preventing and treating inflammatory and autoimmune diseases.

## Introduction

CD4^+^ T cells play crucial roles in the induction of optimal immune responses against pathogenic infections including bacteria, viruses, and malaria parasites by differentiating into effector T helper (Th) cells, such as Th1, Th2, and Th17 cells^1–3^. CD4^+^ T cells are also differentiated into CD4^+^CD25^+^Foxp3^+^ regulatory T (pTreg or iTreg) cells in the periphery^4^. Numerous environmental cytokines and transcription factors involved in the specification of cell lineage commitment have been identified. For example, interferon-γ (IFN-γ)/T-box protein expressed in T cells (T-bet) and interleukin (IL)-4/GATA-binding protein 3 are essential for the development of Th1 and Th2, respectively^5,6^, and transforming growth factor β (TGF β) and IL-6/retinoic acid-related orphan receptor γ t (RORγt) induce Th17 cell lineage commitment^7^. Potentiation of TGFβ signaling in the absence of IL-6 leads to iTreg cell differentiation through the induction of forkhead box (Fox) P3^8^. iTreg cells contribute to optimal immune regulation for suppressing excessive immune responses and preventing autoimmunity in a context-dependent manner^9,10^.

T cell receptor triggering and stimulation with TGFβ and IL-2 increase the expression of Foxp3, a signature marker of Treg cells^11^. Foxp3 transcription is regulated by conserved non-coding DNA sequence and several transcription factors^12,13^. TGFβ-induced Sma and Mad related Family (SMADs) cooperatively interact with nuclear factor of activated T-cells (NFAT) and induce Foxp3 expression through modification of the Foxp3 enhancer element^14^. NFAT and Foxp3 cooperatively upregulate the expression of Treg markers cytotoxic T-lymphocyte-associated antigen 4 (CTLA-4) and CD25^15^. Furthermore, nuclear factor κ B (NF-κB)^16^, FoxOs^17,18^, and runt-related transcription factor 1 (RUNX1)^19,20^ activate Foxp3 expression^17,18^. Nuclear receptor 4A proteins (NR4As) were recently reported to enhance Foxp3 expression in cooperation with RUNX1 and sustain Foxp3 expression in Treg cells^21–23^. Increased Foxp3 subsequently upregulates CD25 expression by cooperation with NFAT and NF-κB^15,24^.

Impressive therapeutic approaches to transplantation, cancer, and autoimmune diseases have been developed based on Treg cell function^25–30^. However, little progress has been made in the development of drugs that promote Treg cell differentiation. Only isoliquiritigenin and naringen isolated from herbal medicine licorice have been shown to promote iTreg cell development and attenuate inflammatory colitis^31^. Researchers are working to isolate novel drugs that increase iTreg cell development and activity to suppress inflammatory diseases. An anti-malarial drug, amodiaquine (AQ) has long been used for treating arthritis^32^ and was recently identified to have potent anti-Parkinsonian potential through activation of NR4A activity and anti-proliferative activity^33,34^.

In this study, we investigated whether AQ could affect iTreg cell development. Our results indicate that AQ promotes iTreg cell development through a significant induction of CD25 and subsequently increases Foxp3 expression, which are controlled by activation of NR4A, and thus suppresses inflammatory colitis, particularly, induced by T cells.

## Results

### Anti-proliferative activity of AQ was diminished in TGFβ-induced iTreg cells

**Figure 1:**
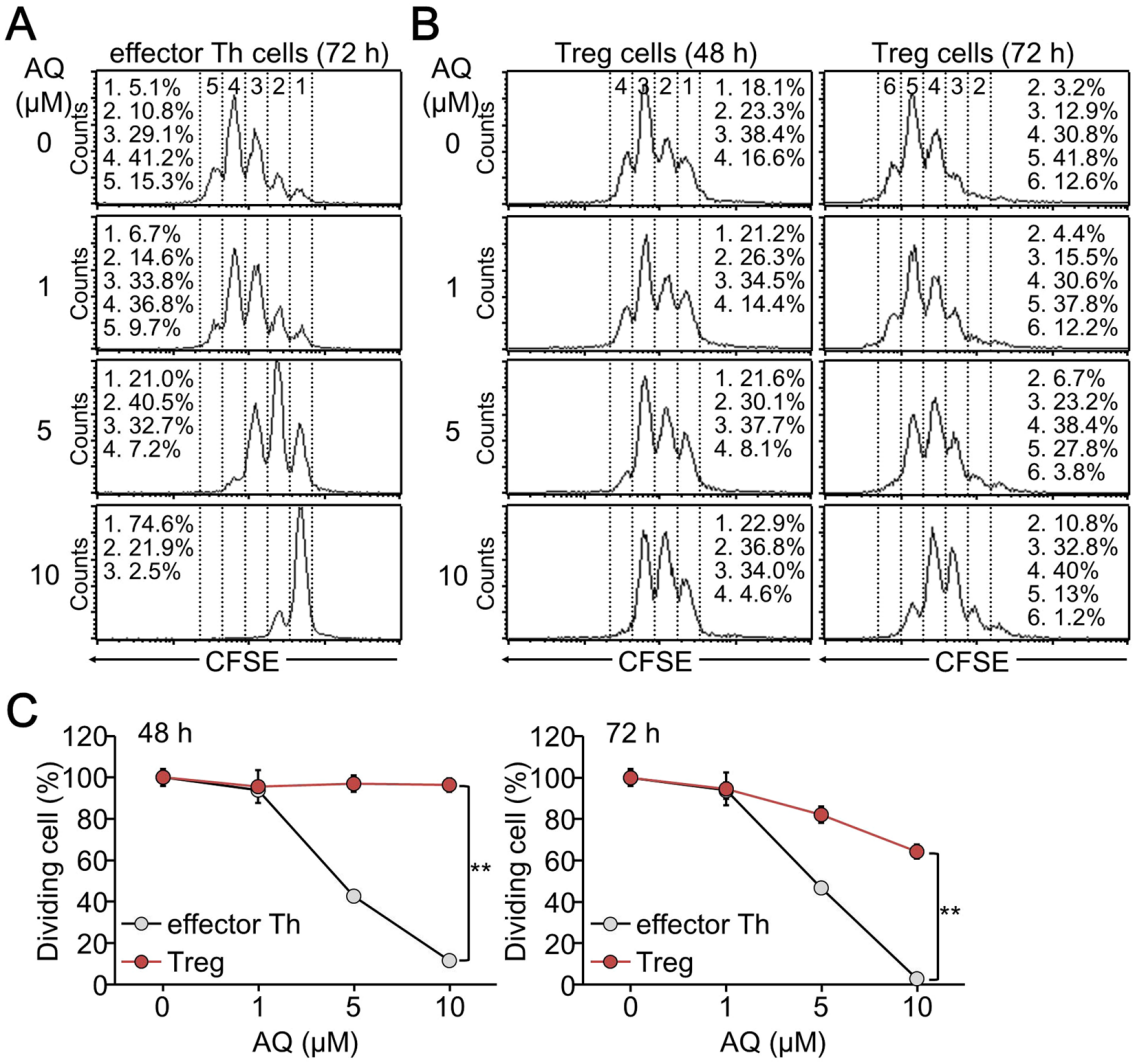
Diminished anti-proliferative activity of AQ in developing iTreg cells. CD4^+^ T cells labeled with CFSE were triggered with anti-TCR and cultured for 72 h under non-skewing (**A**) and for 48 and 72 h under Treg-skewing (**B**) conditions. AQ was added to the cells at 24 h after TCR stimulation. Cells were analyzed by flow cytometry analysis and CellQuest software (**A**,**B**). A representative image of four independent experiments is shown. (**C**) Cell populations of dividing effector T and iTreg cells were calculated at 48 and 72 h under non-skewing and Treg-skewing conditions, respectively, and expressed as mean of four independent experiments. **p < 0.005 and ***p < 0.0005.

To examine the effects of AQ on iTreg cell development, we first examined whether AQ suppressed cell cycle progression under iTreg-skewing conditions. As reported previously^34^, AQ substantially suppressed cell division of developing effector Th cells and dramatically inhibited cell cycle progression under non-skewing conditions. AQ also delayed cell division of T cells under iTreg-skewing conditions, however this inhibitory activity was much decreased when compared to that in effector Th cells (Fig. 1A). Cell populations with higher division numbers were dose-dependently decreased by AQ only in developing effector Th, not iTreg, cells at 48 h after T-cell receptor stimulation (Fig. 1B). At 72 h, AQ moderately decreased the cell population by delaying cell cycle progression. However, the potent anti-proliferative activity of AQ was significantly diminished in dividing iTreg cells (Fig. 1B).

### AQ significantly augmented IL-2 production under Treg-skewing conditions

**Figure 2:**
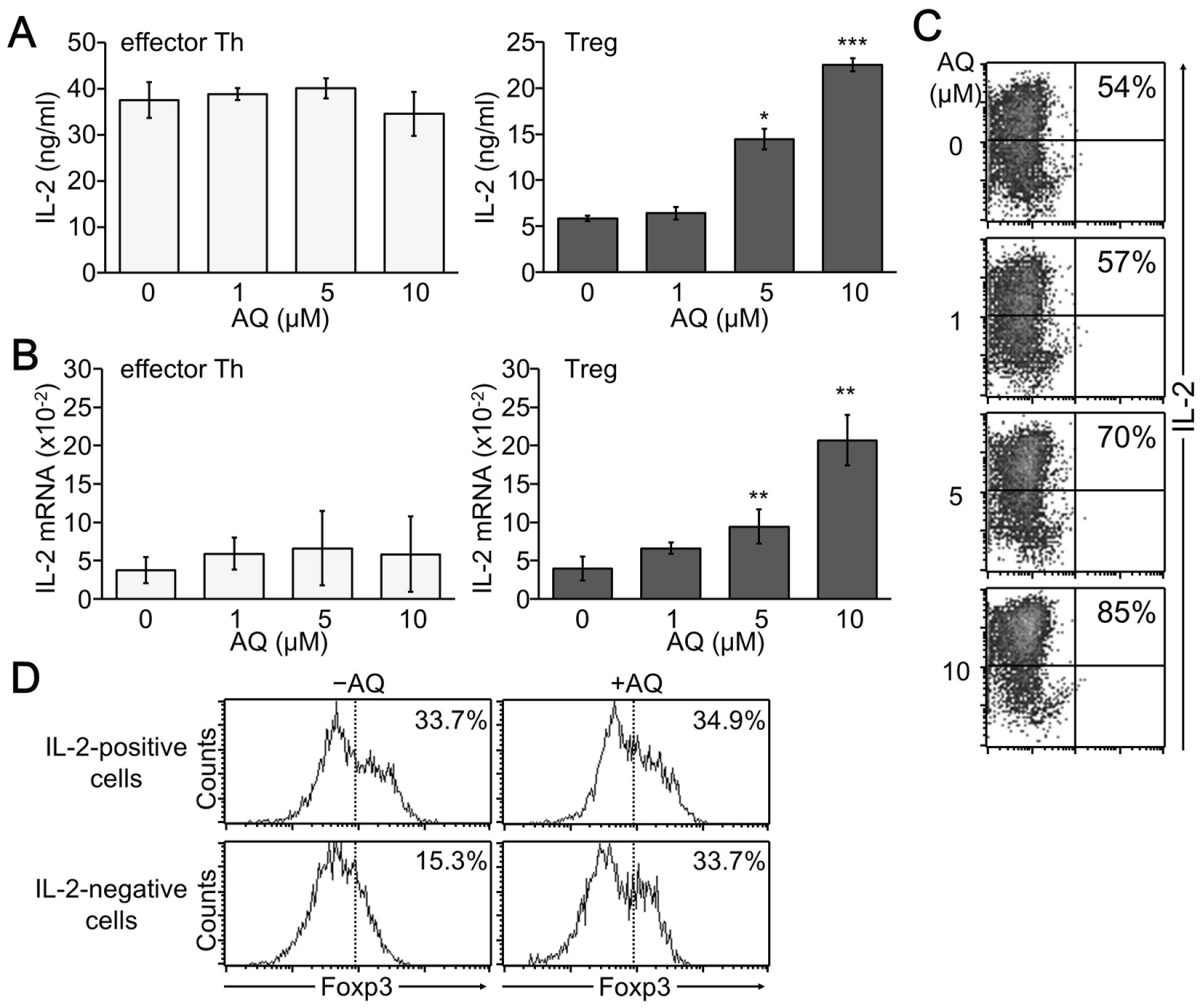
Enhanced IL-2 production by AQ treatment in iTreg cells. CD4^+^ T cells were stimulated and induced to differentiate into effector and iTreg cells. (**A**) Cell supernatants were harvested at day 2 for measuring IL-2 by ELISA. *p < 0.05 and ***p < 0.0005. (**B**) Total RNA was harvested at day 2 and subjected to quantitative real time-PCR analysis of IL-2 transcripts. **p < 0.005. Four independent experiments were performed and similar results were obtained in each. Data shown in A and B represent the mean ± SD of four independent experiments. (**C**) Differentiated iTreg cells were restimulated with PMA and ionomycin for 6 h and stained with PE-conjugated IL-2 followed by flow cytometry analysis. (**D**) Foxp3 expression was analyzed in IL-2-positive and –negative cells of veh- or AQ-treated iTreg cells. A representative image of three independent experiments is shown in C and D.

Because dividing cell populations decreased by treatment with AQ, we then investigated the expression level of IL-2, a T cell growth factor. Although IL-2 production by effector Th cells was not affected by AQ treatment (Fig. 2A and B), IL-2 production by iTreg cells was significantly increased by AQ in a dose-dependent manner (Fig. 2A). Consistently, AQ treatment substantially enhanced IL-2 gene transcription and IL-2-producing cell populations, as confirmed by real time-PCR and intracellular cytokine staining (Fig. 2B and C). Interestingly, IL-2-producing cells expressed higher level of Foxp3 than IL-2-negative cells did. AQ strongly stimulated Foxp3 expression even in IL-2-negative cell populations (Fig. 2D).

### AQ treatment promoted development of CD25^+^Foxp3^+^ iTreg cells

**Figure 3:**
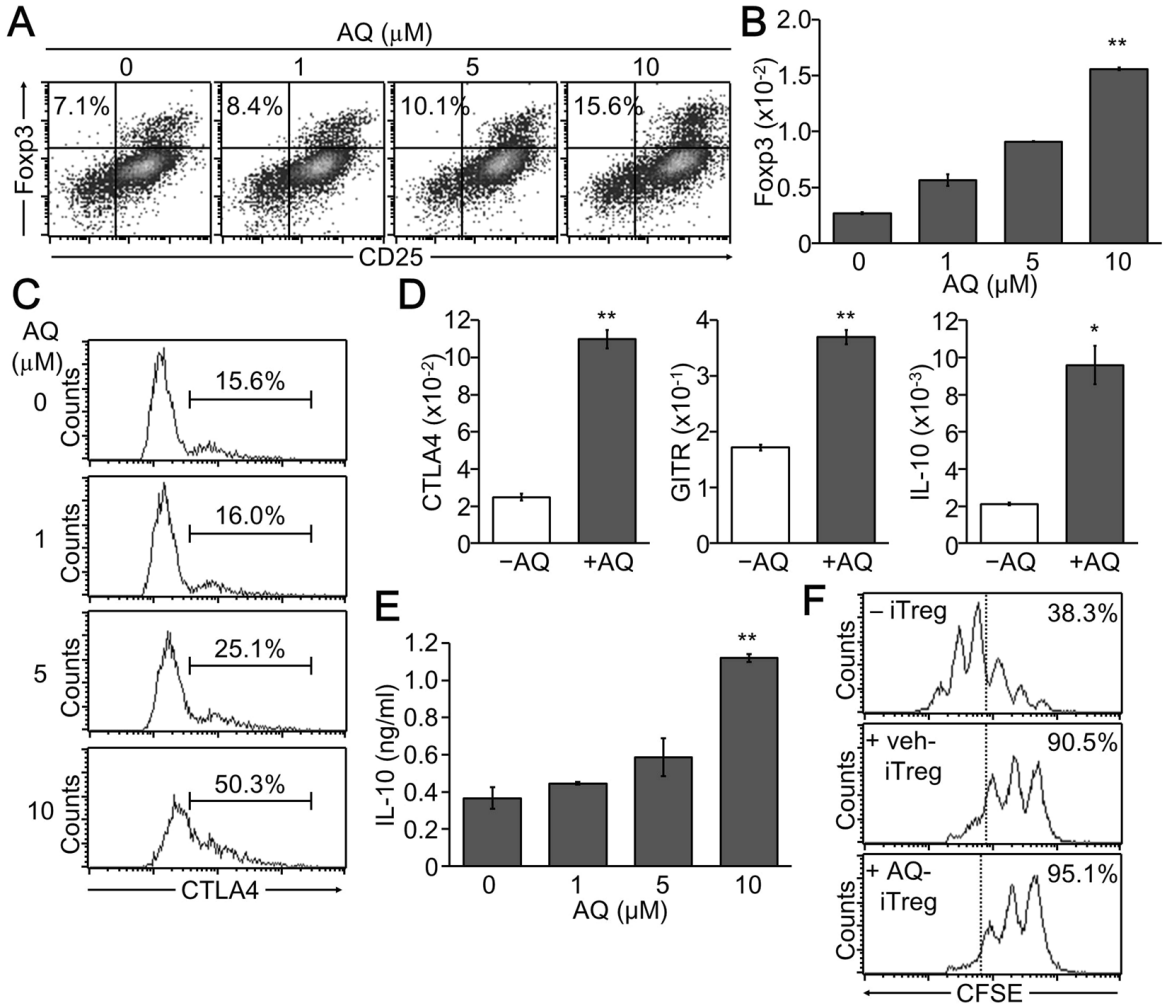
Promoted iTreg cell development by treatment with AQ. CD4^+^ T cells were induced to differentiate into iTreg cells in the presence of AQ. (**A**) Cells were stained with CD25 and Foxp3 antibody and subjected to flow cytometry analysis. A representative experiment of three independent experiments is shown. (**B**) Total RNA was isolated to determine the Foxp3 mRNA level by real time-PCR. Data are the mean of three independent experiments. **p < 0.005. (**C**) iTreg cells were analyzed by staining with CTLA-4. A representative experiment of three independent experiments is presented. (**D**) iTreg cells were harvested for preparing total RNA, and real time-PCR analysis was performed to determine transcript levels of CTLA-4, GITR, and IL-10. *p < 0.05 and **p < 0.005. (**E**) iTreg cells were restimulated with anti-CD3 overnight, and cell supernatants were harvested for IL-10 measurement by ELISA. **p < 0.005. Data are given as mean ± SD of three independent experiments in D and E. (**F**) CFSE-labelled effector T cells were stimulated with anti-CD3 and anti-CD28 in the presence of vehicle- or AQ-treated iTreg cells for 48 h and subjected to flow cytometry analysis. A representative image of three independent experiments is shown.

Because IL-2 production, which is essential for inducing iTreg cell development, was upregulated in AQ-treated iTreg cells, we next assessed the effects of AQ on iTreg cell differentiation. Incubation of CD4^+^ T cells with AQ in the presence of TGFβ increased iTreg cell development, as evidenced by increased expression of CD25 and Foxp3 by AQ treatment (Fig. 3A). AQ treatment significantly and dose-dependently increased relative expression of Foxp3 gene transcript (Fig. 3B). In addition, AQ upregulated the expression of the iTreg cell marker, CTLA-4 at the cell surface (Fig. 3C) and significantly enhanced the mRNA level of Treg cell markers CTLA-4, glucocorticoid-induced tumor necrosis factor receptor-related protein (GITR), and IL-10 (Fig. 3D). Production of IL-10, a signature iTreg cytokine, was enhanced by AQ during Treg cell development (Fig. 3E). We further demonstrated that iTreg cells were functional. Co-culture experiment verified that iTreg cells suppressed cell proliferation of effector T cells and the suppressive activity of AQ-treated iTreg cells were a little stronger than that of control iTreg cells (Fig. 3F and Supple Fig. S1).

### CD25 expression was increased and sustained in AQ-treated iTreg cells

**Figure 4:**
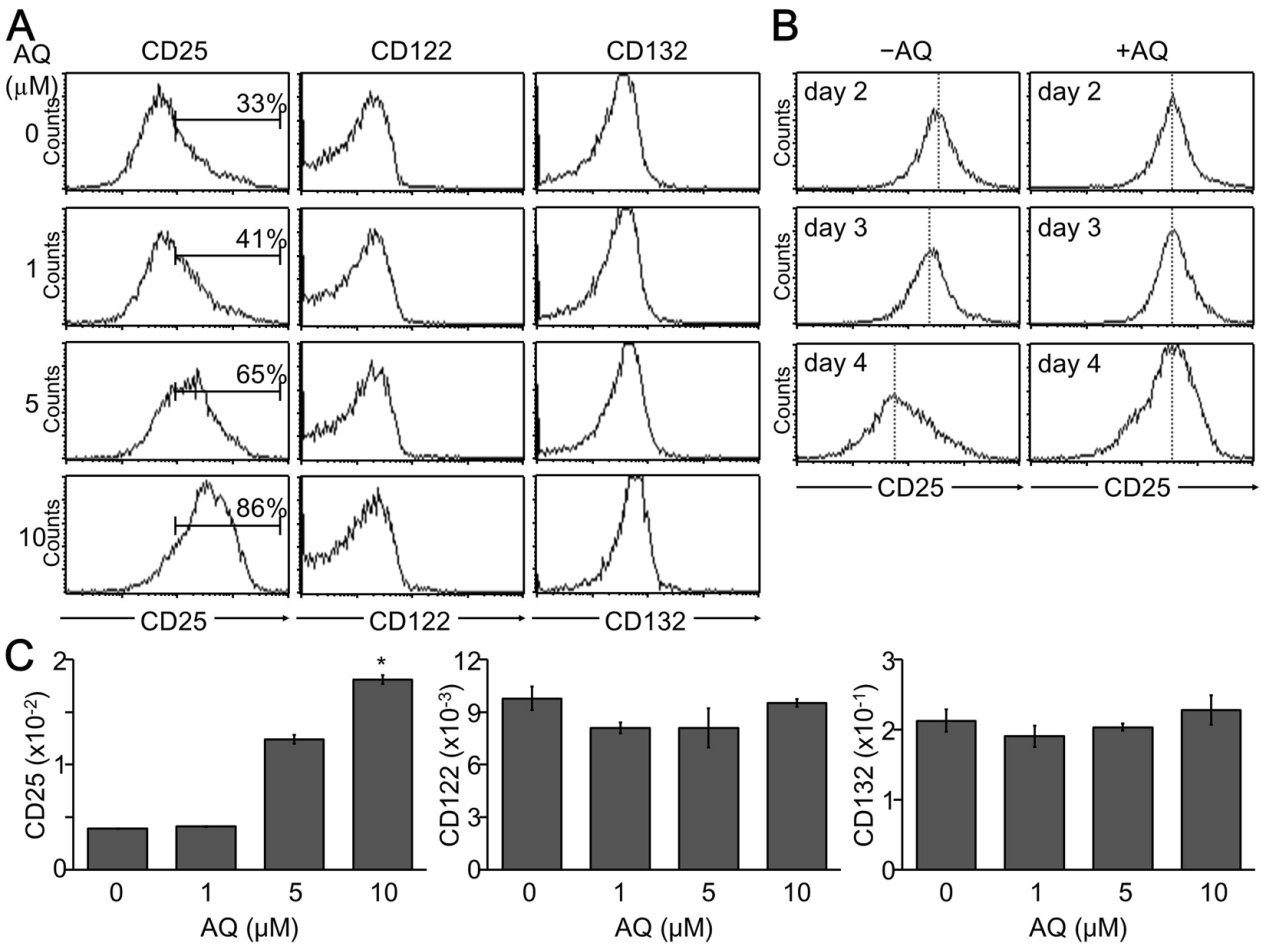
Enhanced and sustained expression of CD25 by the presence of AQ. iTreg cells were developed in the presence of various amounts of AQ. (**A**) iTreg cells were harvested at day 4 post stimulation and stained with antibody against CD25, CD122, or CD132 followed by flow cytometry analysis. (**B**) iTreg cells were incubated with AQ (10 µM) and harvested at days 2, 3, and 4 and subjected to flow cytometry after staining with anti-CD25 (PC61). Three independent experiments were performed and a representative image is shown. (**C**) Total RNA was harvested at day 3 and reversely transcribed into cDNA for subsequent real time-PCR analysis of CD25, CD122, and CD132. Data are given as mean from three independent experiments. *p < 0.05.

Not only Foxp3 but also CD25 expression was elevated by AQ treatment during iTreg cell development. We thus examined whether AQ directly affected CD25 expression. CD25 (IL-2 receptor α, IL-2R) expression was dose-dependently upregulated at the cell surface after treatment with AQ, whereas CD122 (IL-2Rβ) and CD132 (γ common chain) were not changed (Fig. 4A). Interestingly, CD25 expression that was increased upon TCR stimulation decreased at a later stage of iTreg cell development, but AQ treatment sustained the expression level (Fig. 4B). In accordance with the increased CD25 protein expression, the relative mRNA level of CD25 was upregulated by AQ. However, AQ did not affect the mRNA level of CD122 and CD132 (Fig. 4C). Interestingly, AQ-induced CD25 expression was not observed in the isolated peripheral Treg cells but was prominent in developing iTreg cells (Supple Fig. S2).

### Upregulated CD25 expression by AQ was independent of IL-2/IL-2R signaling

**Figure 5:**
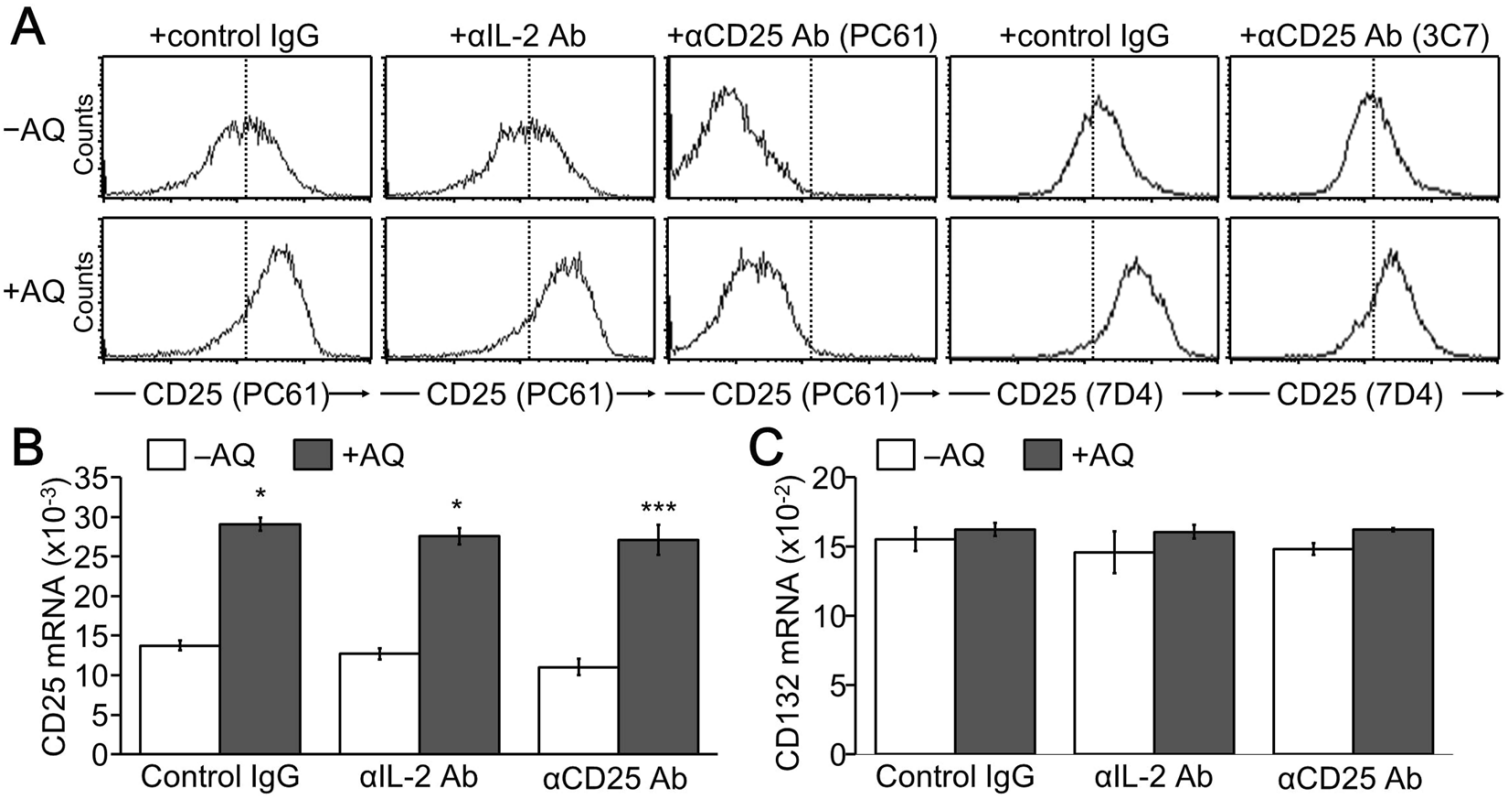
IL-2/IL-2R-independent CD25 induction by AQ. CD4^+^ T cells were induced to differentiate into iTreg cells and incubated with AQ in the presence of control IgG, IL-2 neutralizing antibody (BD Pharmingen, S4B6), or IL-2R blocking antibody (PC61 or 3 C7) for 2 days before harvest. (**A**) Cells were harvested at day 3 and incubated with anti-CD25 antibody (PE-PC61 or FITC-7D4) followed by flow cytometry analysis. A representative experiment of three independent experiments is shown. (**B**,**C**) Total RNA was prepared with TRIzol and subjected to reverse transcription and quantitative real time-PCR analysis. *p < 0.05 and ***p < 0.0005. Relative transcript levels of CD25 (**B**) and CD132 (**C**) were determined after normalization to the actin level from three independent experiments.

To clarify whether AQ-mediated CD25 induction was due to the activation of IL-2/IL-2R signaling, we investigated the expression level of CD25 in the presence of anti-IL-2 and anti-CD15 antibodies. AQ treatment increased the surface expression level of CD25 in iTreg cells, which was not affected by neutralization of IL-2 with anti-IL-2 antibody (Fig. 5A). We blocked CD25 with blocking antibodies and analyzed cell surface CD25 expression after treatment with AQ. Cell surface CD25 expression was still enhanced by AQ in the presence of CD25 blocker (Fig. 5A). In addition, AQ significantly elevated CD25 mRNA expression regardless of IL-2 neutralization and CD25 blockade, but did not affect CD132 gene transcription (Fig. 5B and C).

### AQ activated CD25 transcription through activation DNA binding activity of NR4A

**Figure 6:**
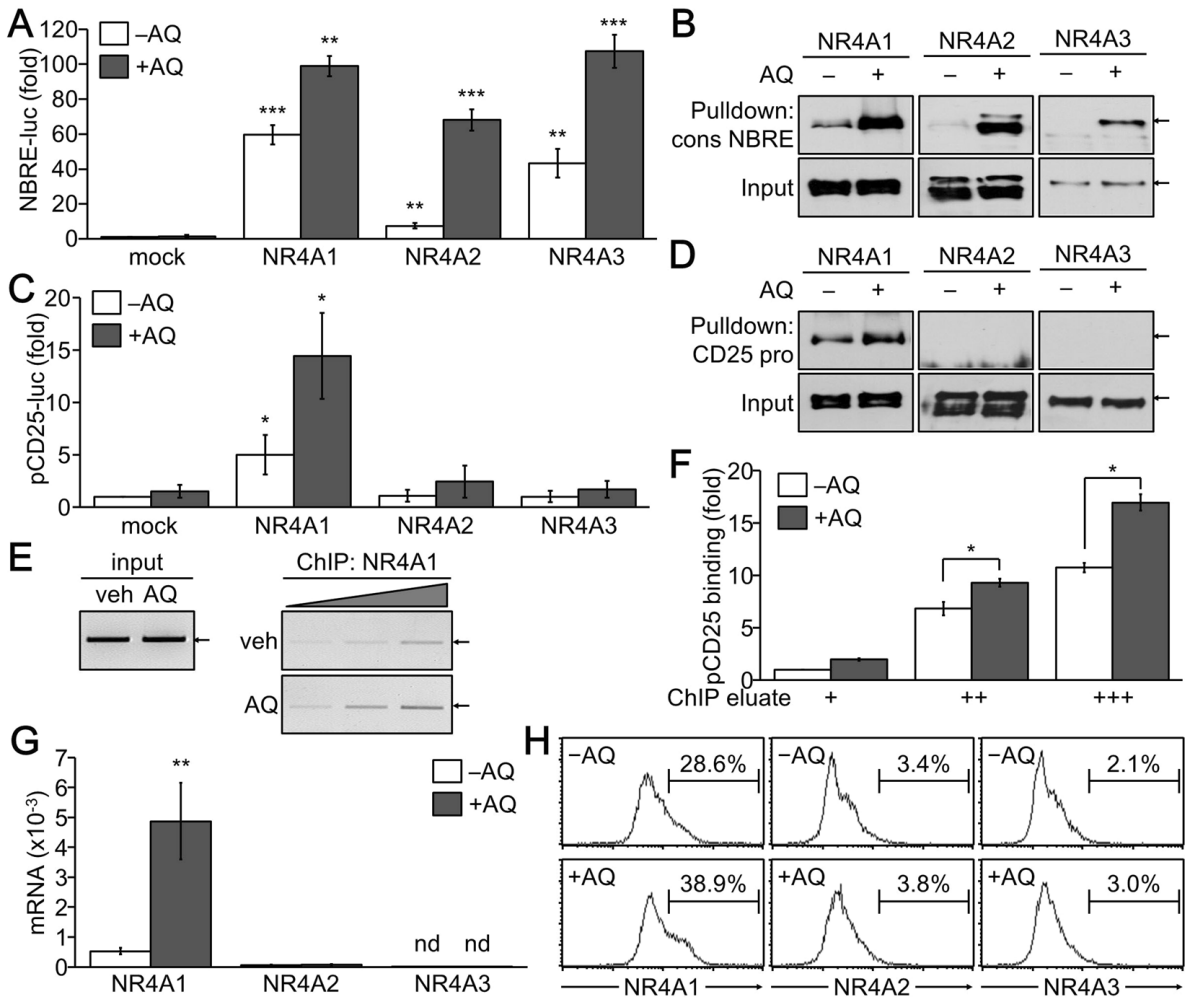
Increased NR4A1-induced CD25 transcription by AQ. (**A**) Highly transfectable 293T cells were transfected with NBRE-luc reporter and NR4As expression vector and subsequently treated with either vehicle or AQ (50 µM) for 24 h. Cells were harvested for the reporter assay. Relative luciferase activity was calculated from three independent experiments and expressed as fold induction compared to mock control. **p < 0.005 and ***p < 0.0005. (**B**) NR4As proteins were overexpressed in 293T cells, which were then incubated with AQ for 24 h. Cell proteins were incubated with biotinylated consensus NBRE, followed by precipitation with streptavidin-conjugated agarose beads. DNA-proteins complexes were resolved by SDS-PAGE and analyzed by immunoblotting with antibodies against NR4A1, NR4A2, and NR4A3. (**C**) 293T cells were transfected with CD25 promoter reporter gene with NR4As expression vector followed by AQ treatment. Reporter activity is given as mean ± SD of three independent experiments. *p < 0.05. (**D**) 293T cells were exogenously expressed with NR4A proteins and incubated with AQ for 24 h. Cell proteins were incubated with NBRE within the CD25 gene promoter, resolved by SDS-PAGE, and analyzed by immunoblotting with antibodies against NR4A1, NR4A2, and NR4A3. (**E**–**H**) CD4^+^ T cells were induced iTreg in the presence of AQ (10 µM) for 3 days and harvested. For Chip, cell extracts were incubated with an NR4A1 antibody and the chromatin-protein complex was precipitated followed by semi-quantitative PCR (**E**). Relative level of CD25 promoter bound to NR4A1 was quantitated by real time-PCR (**F**). Total RNA was harvested and relative expression levels of NR4A1, NR4A2, and NR4A3 were determined after normalization to the actin level (n = 3) **p < 0.005. (**G**) Cells were permeabilized and incubated with antibodies against NR4A1, NR4A2, and NR4A3, followed by flow cytometry and CellQuest analysis (**H**). A representative experiment of three independent experiments is presented in B, D, E, and H.

We attempted to explain the molecular mechanisms underlying the IL-2/IL-2R-independent induction of CD25 by AQ. Recently, AQ was identified as a novel NR4A ligand that activates NR4A-mediated gene transcription^33^. We confirmed that AQ treatment further enhanced the NR4A-binding responsive element (NBRE)-linked reporter activity that was increased by the overexpression of NR4A proteins NR4A1, NR4A2, or NR4A3 (Fig. 6A). DNA-binding activity of NR4As was markedly increased by AQ (Fig. 6B). Furthermore, the CD25 promoter activity was increased by the overexpression of NR4A1, which was further elevated by treatment with AQ (Fig. 6C). Accordingly, we found that AQ enhanced the binding of NR4A1 to the putative NBRE within the CD25 gene promoter (Fig. 6D). Interestingly, AQ had no significant effect on NR4A2 and NR4A3, which failed to induce the CD25 promoter activity and bind to the CD25 promoter (Fig. 6C,D). More critically, chromatin immunoprecipitation and quantitative real time-PCR verified that endogenous NR4A1 directly bound to the CD25 gene promoter in iTreg cells, and AQ stimulated complex formation between NR4A1 and CD25 promoter (Fig. 6E,F). We additionally found that the expression level of NR4A1, not NR4A2 nor NR4A3, was increased by AQ at the mRNA and protein levels as well (Fig. 6G,H).

### AQ suppressed inflammatory colitis development *in vivo*

**Figure 7:**
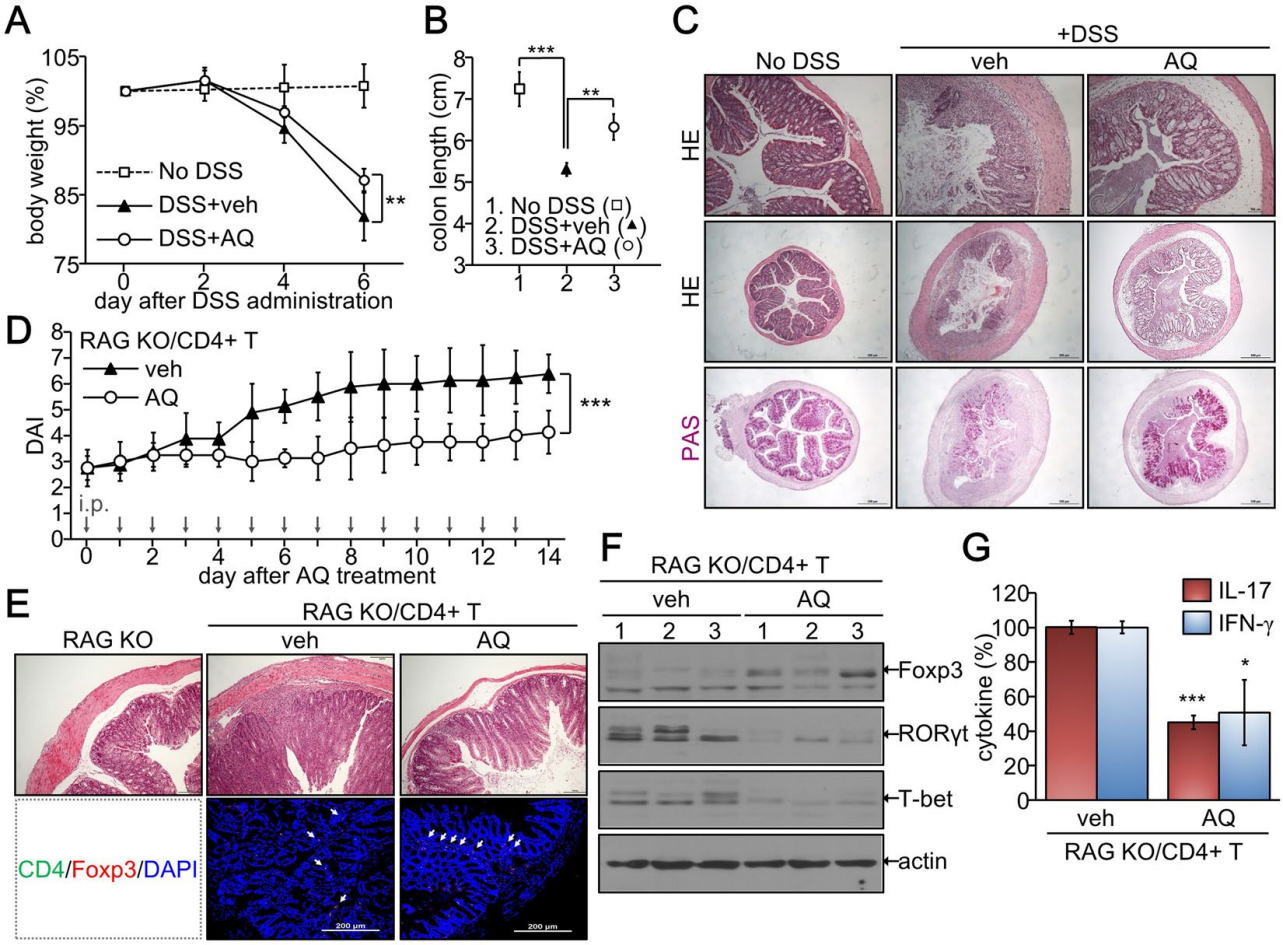
Attenuation of colitis by AQ *in vivo*. (**A**–**C**) C57BL6 mice were administered with normal water (no DSS, n = 6) or 3% DSS water (DSS, n = 12) for 6 days. DSS-treated mice were injected with either vehicle (DSS + veh, n = 6) or AQ (10 mg/kg, DSS + AQ, n = 6) every day. Three independent experiments were performed for statistical analysis. Body weight (n = 15 per group) was measured and weight loss was calculated compared to that on day 0. **p < 0.005 (**A**). Colon length was determined and expressed as mean ± SD of 6 mice. **p < 0.005 and ***p < 0.0005 (**B**). Representative images of HE and PAS staining of colon sections (n = 6) (**C**). (**D**–**G**) Naïve CD4^+^ T cells were isolated from C57BL6 mice and adoptively transferred to RAG KO mice (RAG KO/CD4^+^ T, n = 12). After 4 weeks, mice were injected daily with vehicle (n = 6) or AQ (10 mg/kg, n = 6) for 2 weeks. This experiment was repeated twice and DAI (n = 12 per group) was determined by observing the weight loss, diarrhea, and rectal bleeding. ***p < 0.0005 (**D**). Colon tissue sections (n = 6 per group) were stained with HE or fluorescence-tagged antibodies against CD4 and Foxp3. DPI stained nuclei and representative images are shown (**E**). Total proteins (n = 3) were harvested from spleen and subjected to immunoblotting analysis of FoxP3, RORγt, T-bet, and β-actin. Representative blots of three independent experiments are shown (**F**). Colon tissues (n = 6) were excised and cultured in DMEM for 24 h, and cell supernatants were used for determining IL-17 and IFN-γ by ELISA. *p < 0.05 and ***p < 0.0005 (**G**).

To confirm the immune suppression by AQ *in vivo*, mice were fed DSS and intraperitoneally injected with either vehicle or AQ. Body weight loss and colon shortening were induced in the DSS-treated group, but these changes were attenuated in the AQ-treated group (Fig. 7A,B). Histological examination demonstrated that AQ reduced the immune cell infiltration and inhibited epithelial cell destruction with goblet cell depletion in the DSS-induced colitis model (Fig. 7C). Furthermore, T cell-induced colitis in recombinase-activating gene (RAG) knockout (KO) mice was diminished by administration with AQ, as demonstrated by the decreases in disease activity index and histopathology (Fig. 7D,E). Foxp3+ Treg cells were more frequently found in AQ-treated colon tissues (Fig. 7E). Consistently, AQ administration increased the expression of Foxp3, but decreased the expression of inflammatory markers RORγt and T-bet (Fig. 7F). Moreover, the inflammatory cytokines IL-17 and IFN-γ produced by inflammatory T cells were significantly diminished in the AQ-treated group (Fig. 7G).

## Discussion

Our study demonstrated that AQ functioned as an activator of CD25^+^Foxp3^+^ Treg cell development through a significant induction of CD25 expression. AQ-mediated CD25 induction was independent of the IL-2/IL-2R signaling pathway, but depended on NR4A1 activity. Moreover, AQ significantly attenuated colonic inflammation and increased Treg cell generation *in vivo*. Our results suggest that AQ is beneficial for controlling inflammatory and autoimmune diseases by promoting Treg cell generation.

AQ substantially suppressed T cell proliferation through a significant induction of p21 expression^34^. However, the anti-proliferative function of AQ was substantially diminished in TGFβ-induced iTreg cells. The cell division numbers with different carboxyfluorescein succinimidyl ester (CFSE) intensity decreased in AQ-treated effector Th cells. In iTreg cells, however, cell cycle progression was sustained in the presence of AQ, although the overall growth rate of iTreg cells was delayed. Interestingly, IL-2 production was significantly elevated in iTreg cells by AQ. Because IL-2 is essential for inducing and expanding iTreg cells through IL-2R-mediated signal transducer and activator of transcription (STAT) 5 activation^35^, the increase of IL-2 by AQ would promote iTreg cell development and expansion through its receptor. Further analysis revealed that AQ also raised the expression of CD25, an IL-2Rα, at the transcriptional level, but AQ-induced CD25 expression was not inhibited by the presence of IL-2 and IL-2R blockers. These results suggest that induction of IL-2 and CD25 expression by AQ requires the activation of specific transcription factors in response to AQ, not IL-2/IL-2R-mediated signaling molecules. Indeed, AQ activated DNA-binding and transcriptional activity of NR4As by binding to the ligand-binding domain of NR4As, thereby increasing NR4A-mediated CD25 expression. The fact that Foxp3 expression is increased by NR4As in Treg cells^21^ supports a critical finding that AQ promotes Treg cell development through activation of NR4A-induced CD25 and Foxp3 expression. However, it remains to be clarified whether AQ induces IL-2 expression in iTreg cells through NR4A activation or not.

AQ activates the DNA-binding and transcriptional activity of three isoforms of the NR4A family at comparable levels. However, CD25 promoter activity is distinctively and significantly increased by NR4A1, which is further markedly enhanced by AQ treatment. Both NR4A2 and NR4A3 fail to increase the CD25 promoter activity and show no cooperative activity with AQ in CD25 expression. Thus, it will be important to understand how the CD25 promoter is selectively recognized by NR4A1, not NR4A2 or NR4A3, and whether NR4A1 cooperates with other Treg-specific transcription factors for inducing CD25 transcription. Interestingly, the relative NR4A1 transcript level was higher than that of NR4A2 or NR4A3 in Treg cells and was further significantly increased by treatment with AQ. NR4A1 contains consensus NBRE in its promoter, and its transcription is regulated by autoactivation by binding with an agonist like cytosporone B^36,37^. Therefore, our findings suggest that AQ binding to NR4A1 specifically increases NR4A1 gene transcription and further activates CD25 and Foxp3 expression, thereby promoting iTreg cell development. Future work is needed to identify whether other NR4A ligands would affect the expression of CD25 and Foxp3 and iTreg cell development and have beneficial effects on chronic inflammatory and autoimmune diseases.

## Materials and Methods

### Materials

AQ (4-([7-Chloro-4-quinolinyl]amino)-2-([diethylamino]methyl)phenol, MW = 464.81, purity >98%) was purchased from Sigma-Aldrich (St. Louis, MO). AQ was dissolved in 100% DMSO and then diluted in culture media. Vehicle control was treated with 0.1% DMSO. All cytokines and antibodies against cytokines were purchased from BioLegend (San Diego, CA), eBioscience (Thermo Fisher Scientific, Carlsbad, CA), and BD Pharmingen (BD Biosciences, San Jose, CA).

### Mice

C57BL6 (8–10 weeks of age, male) and RAG2 KO (6 weeks of age, male) mice were housed in a specific pathogen-free animal facility of Ewha Womans University. All animal handling and experiments were approved by the Institutional Animal Care and Use Committee (2012-01-071, 2014-01-011) and conducted in accordance with the institutional, national, and international laws and guidelines.

### *In vitro* CD4^+^ T cell culture and proliferation assay

Mouse CD4^+^ T cells were isolated from the lymph node and spleen using naive CD4^+^ T cell isolation kit (Miltenyi Biotec, Auburn, CA) and stimulated with plated-bound anti-CD3 (2 µg/ml, BioLegend 145-2C11) and anti-CD28 (1 µg/ml, BD Pharmingen, 37.51). Cells were additionally stimulated with recombinant human IL-2 (10 U/ml, BD Pharmingen) and then cultured either under non-skewing (without additive cytokines) or Treg-skewing (5 µg/ml anti-IL-4, 5 µg/ml anti-IFN-γ, and 5 ng/ml TGFβ, BioLegend) conditions^38,39^ in the absence or presence of AQ. For the cell proliferation assay, CD4^+^ T cells were labeled with 5 µM CFSE (Sigma-Aldrich) and stimulated with plate-bound anti-CD3 (2 µg/ml) and anti-CD28 (1 µg/ml) antibodies for 24 h. Cells were then treated with AQ or incubated with iTreg cells treated with AQ for 3 days for an additional 24 or 48 h and harvested for flow cytometry analysis^34^.

### Reverse transcription and real time-PCR

Total RNA was prepared with TRIzol reagent (Invitrogen, Carlsbad, CA) and reversely transcribed to cDNA using reverse transcriptase (Promega, Madison, WI). Quantitative real time-PCR was performed using the StepOnePlus system (Applied Biosystems Inc., Foster City, CA). Specific primers were as follows; 5′-cctgagcaggatggagaattaca-3′ and 5′-tccagaacatgccgcagag-3′ for IL-2; 5′-cgttgcttaggaaactcctgga-3′ and 5′-gctttctcgatttgtcatggg-3′ for CD25; 5′-cgtccatgccaagtcgaac-3′ and 5′-gatgcctgcctcacaagactt-3′ for CD122; 5′-ggttggaacgaatgcctccaattc-3′ and 5′- gcagaaccgttcactgtagtctgg-3′ for CD132; 5′-gggctcagttgcataaaccga-3′ and 5′- acactgccagcttttggtttcc-3′ for CTLA-4; 5′-aaggttcagaacggaagtg-3′ and 5′-gggtctccacagtggtact-3′ for GITR; 5′-tttgaattccctgggtgagaa-3′ and 5′-acaggggagaaatcgatgaca-3′ for IL-10; 5′-gttgatgttcccgcctttg-3′ and 5′-cctggagcccgtgtcg-3′ for NR4A1; 5′-gctgccctggctatggt-3′ and 5′-caggtagttgggtcggtt-3′ for NR4A2; 5′-cactgatctccccaaagaag-3′ and 5′-gcaggacaagtccattgc-3′ for NR4A3; 5′-agagggaaatcgtgcgtgac-3′ and 5′-caatagtgatgacctggccgt-3′ for actin. Relative transcripts were calculated after normalization to the level of actin.

### Immunoblot analysis

Total proteins were extracted from developing iTreg cells or spleen with RIPA lysis buffer and resolved by SDS-PAGE followed by transfer to polyvinylidene difluoride membranes. Protein blots were incubated with specific antibodies against NR4A1, NR4A2, NR4A3, Foxp3 (Cell Signaling Technology, Beverly, MA), RORγt, T-bet, and actin (Santa Cruz Biotechnology, Santa Cruz, CA) and subsequently incubated with peroxidase-conjugated secondary antibody. Protein bands were detected by enhanced chemiluminescent reaction and autoradiography.

### Cytokine measurement and flow cytometry

For determining cytokines by enzyme-linked immunosorbent assay (ELISA), cell supernatants were collected from developing iTreg cells and incubated with antibodies against IL-2, IL-17, IFN-γ, and IL-10 in enzyme immunoassay plates (BD Pharmingen). Colorimetric changes were measured using a microplate reader (Molecular Devices, Sunnyvale, CA). For intracellular cytokine staining, cells were stimulated with 12-phorbol 13-myristate acetate (PMA) and ionomycin for 6 h or anti-CD3 (1 µg/ml) overnight and pre-treated with monensin (4 µM) for 2 h before harvest. Cells were permeabilized and incubated with PE-conjugated anti-IL-2 antibody (BioLegend, JES6-5H4) followed by flow cytometry and CellQuest analysis (BD Biosciences). iTreg cells were incubated with anti-CD25 antibody (PC61, BD Pharmingen or 3C7, BioLegend) and analyzed with antibodies against CD25 (PC61 or 7D4, BD Pharmingen) and FoxP3 (eBioscience, FJK-16s). For cell surface marker staining and flow cytometry, cells were fixed and incubated with antibodies against CTLA-4 (UC10, BD Pharmingen), CD25 (PC61 or 7D4), CD122 (TM-beta1), and CD132 (TUGm2) and subjected to flow cytometry and CellQuest analysis.

### Reporter gene assay

Highly transfectable 293T cells were transiently transfected with expression vector for NR4A1, NR4A2, or NR4A3 and reporter genes pCMVβ (Promega) and either NBRE-luc^33^ or pCD25-luc. The pCD25-luc contains the −600 to +10 region of the mouse CD25 promoter region linked to the pGL2-basic reporter gene (Promega). Relative luciferase activity was measured using a luciferase assay kit (Promega) and normalized with β-galactosidase activity (Galacto-light system, Thermo Fisher Scientific) of pCMVβ.

### DNA pulldown assay

293T cells were transfected with expression vector for NR4A1, NR4A2, and NR4A3 and cultured for 48 h. Total protein lysates were extracted in HKMG (10 mM HEPES pH 7.9, 100 mM KCl, 5 mM MgCl_2_, 1 mM DTT, 0.1% NP-40, and 10% glycerol) buffer and incubated with biotinylated double-stranded DNA of either consensus NBRE or putative NBRE of mouse CD25 promoter for 1 h. The protein-DNA binding complex was precipitated with streptavidin-agarose beads and the subjected to SDS-PAGE and immunoblotting analysis.

### Chromatin immunoprecipitation and quantitative analysis

Chromatin immunoprecipitation (ChIP) was performed with Magna ChIP^TM^ A/G kit according to the manufacturer’s instructions (Millipore, Billerica, MA). Briefly, iTreg cells induced in the presence of either veh or AQ for 3 days were fixed in 1% formaldehyde and subjected to cell lysis and sonication. The sonicated chromatin samples were incubated with anti-NR4A1 antibody overnight and subsequently with protein A/G agarose beads for 1 h. Immune complexes were washed with wash buffer three times. DNA was eluted by adding elution buffer and used for quantitative real time-PCR and semi-quantitative PCR analysis using serial dilutions of DNA eluates.

### Colitis development and histological examination

C57BL6 mice (10–12 weeks of age, male, n = 18) were administered with 3% dextran sulfate sodium (DSS; MP Biomedicals, Solon, OH) water for 6 days and were also injected daily with AQ (10 mg/kg, i.p.). Body weight, stool consistency, and blood in the stool were monitored daily, and colon length was measured at sacrifice. Colon tissue sections were prepared and stained with hematoxylin & eosin (HE) and periodic acid Schiff (PAS) staining solution. Colon tissues were incubated with antibodies against CD4 and Foxp3 and subsequently Alexa 488- or Alex555–conjugated secondary antibodies, followed by confocal fluorescence microscopy observation.

### Establishment of T cell-induced colitis and colon tissue culture

Naïve CD4^+^ T cells were isolated from C57BL6 mice using a naïve T cell enrichment kit (R&D Systems, Minneapolis, MN) and transferred to RAG KO mice (6 weeks of age, male) by intraperitoneal injection. Four weeks later, mice were administered with vehicle or AQ (10 mg/kg, daily, i.p.) for 2 weeks. Mice were monitored every day, and disease activity index (DAI) was determined by scoring percent weight loss, diarrhea score, and rectal bleeding score. Colon tissues (1 cm) were excised, washed with PBS, and cultured in complete DMEM for 24 h. Supernatants were harvested and subjected to ELISA to determined IL-17 and IFN-γ.

### Statistical analysis

All data are given as mean ± SD of at least three independent experiments. Statistical significance was determined using a one-way analysis of variance (ANOVA) and was indicated as follows: *p < 0.05; **p < 0.005; and ***p < 0.0005.

## Electronic supplementary material

Supplementary Figure
