# Supplementary material for: Enhanced CD25+Foxp3+ regulatory T cell development by amodiaquine through activation of nuclear receptor 4A
Source: Sci Rep. 2017 Dec 5;7:16946. doi: 10.1038/s41598-017-17073-y (PMC5717225; doi:10.1038/s41598-017-17073-y)

Enhanced CD25$^+$Foxp3$^+$ regulatory T cell development by amodiaquine through activation of nuclear receptor 4A

Hee Yeon Won[1], Ji Hyun Shin[1], Sera Oh[1], Hana Jeong[1], and Eun Sook Hwang[1*]

[1]College of Pharmacy and Graduate School of Pharmaceutical Sciences, Ewha Womans University, Seoul 03760, Korea

Supplementary Fig S1. Suppression of effector T cell proliferation by iTreg cells.

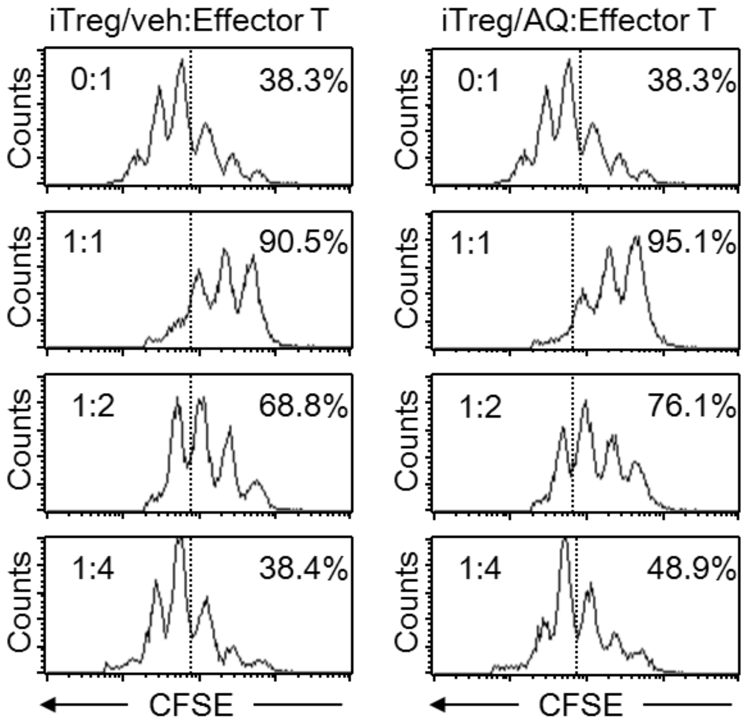

Supplementary Fig S1. Induction of CD25 expression by AQ in developing iTreg cells but not in the isolated peripheral Treg cells.

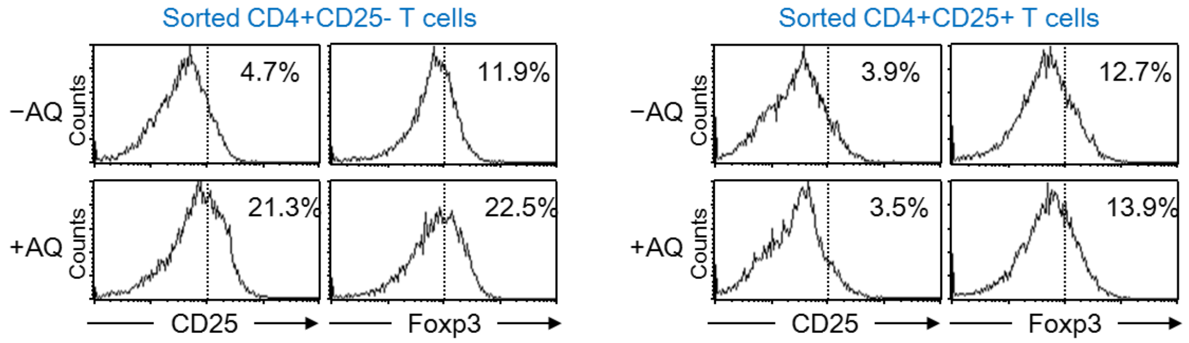

Supplementary Fig. S3. Uncropped blots in Fig. 6B

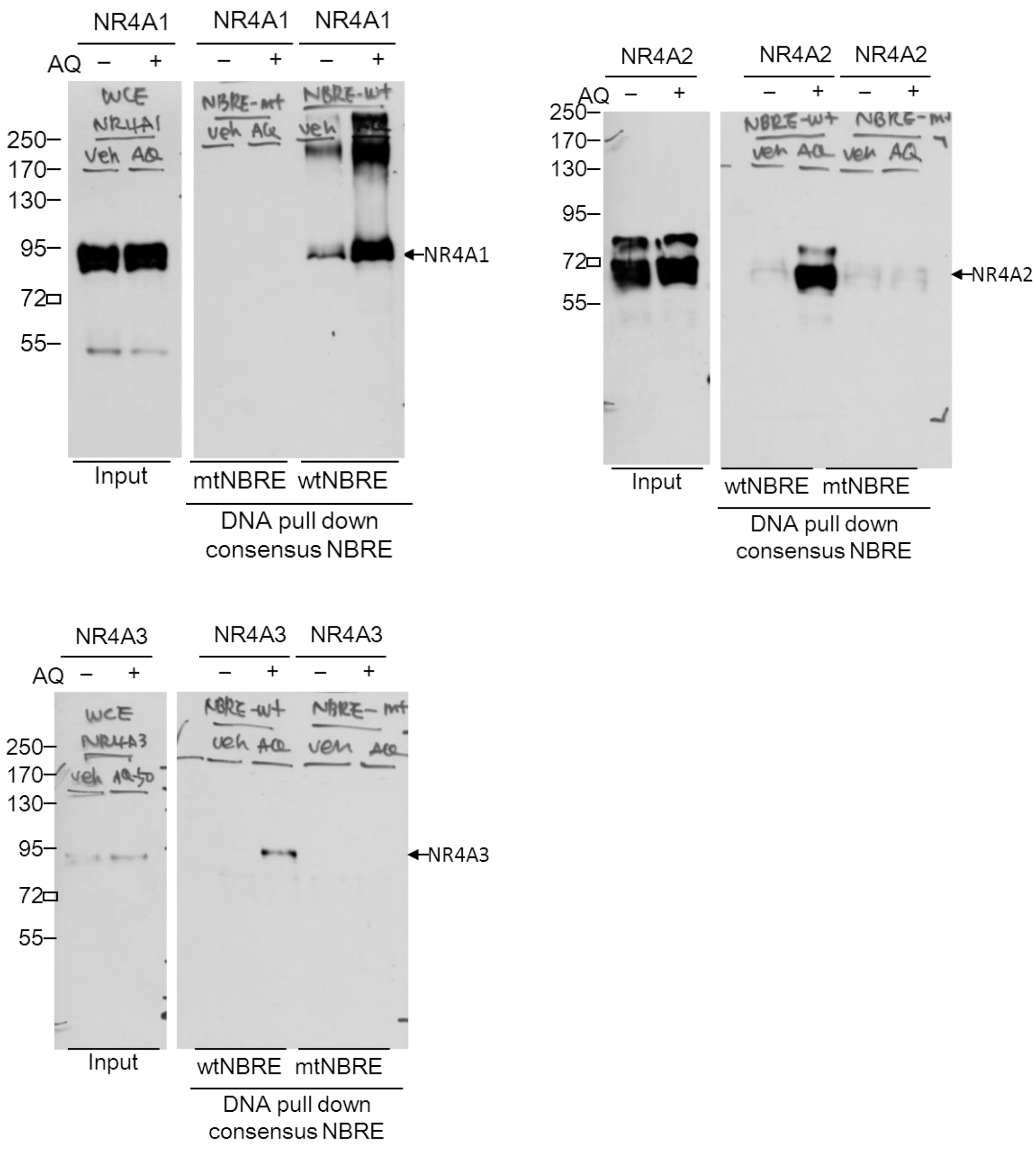

Supplementary Fig. S4. Uncropped blots in Fig. 6D

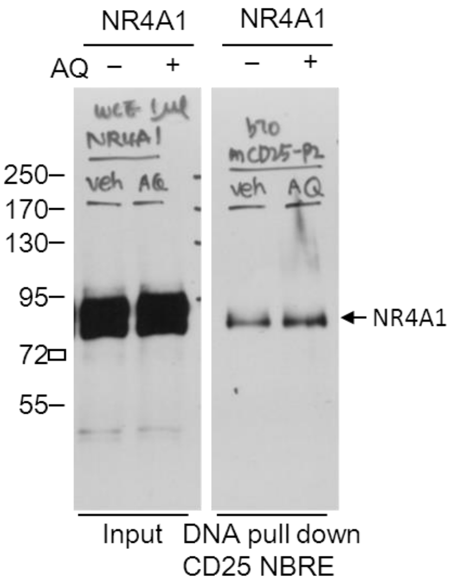

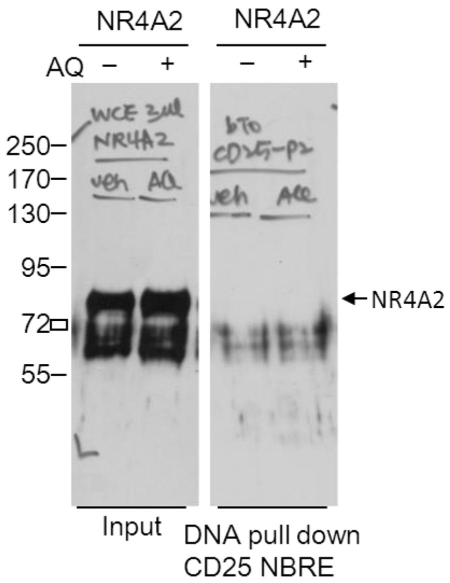

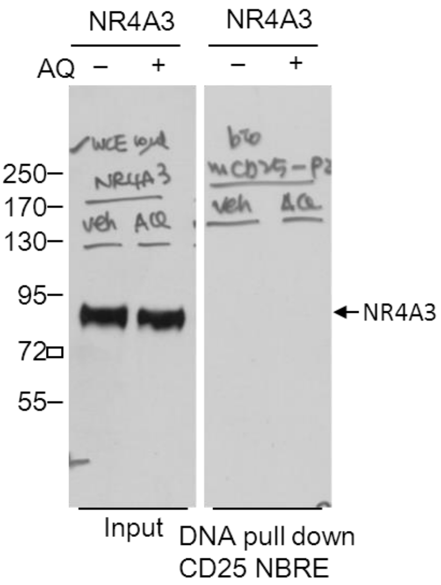

Supplementary Fig. S5. Uncropped blots in Figure 6E

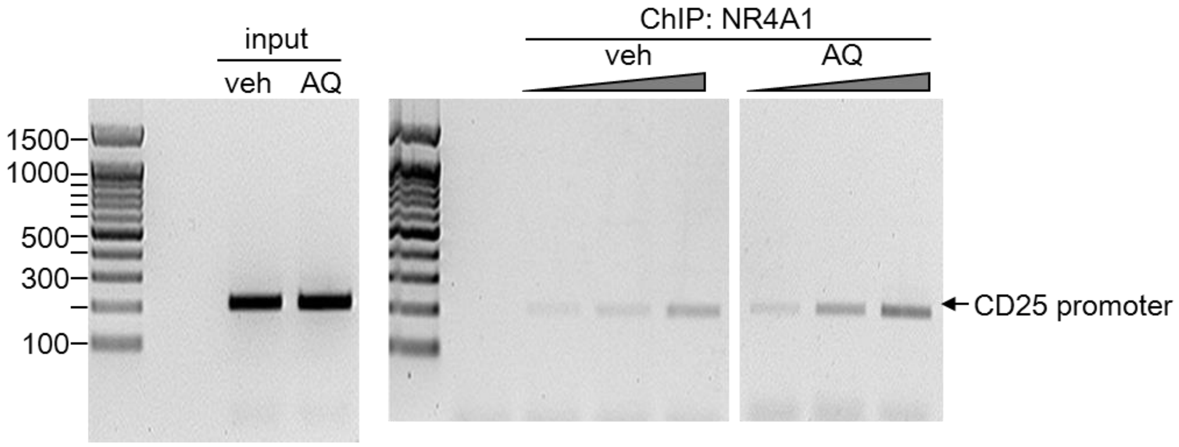

Supplementary Fig. S6. Uncropped blots in Figure 7E

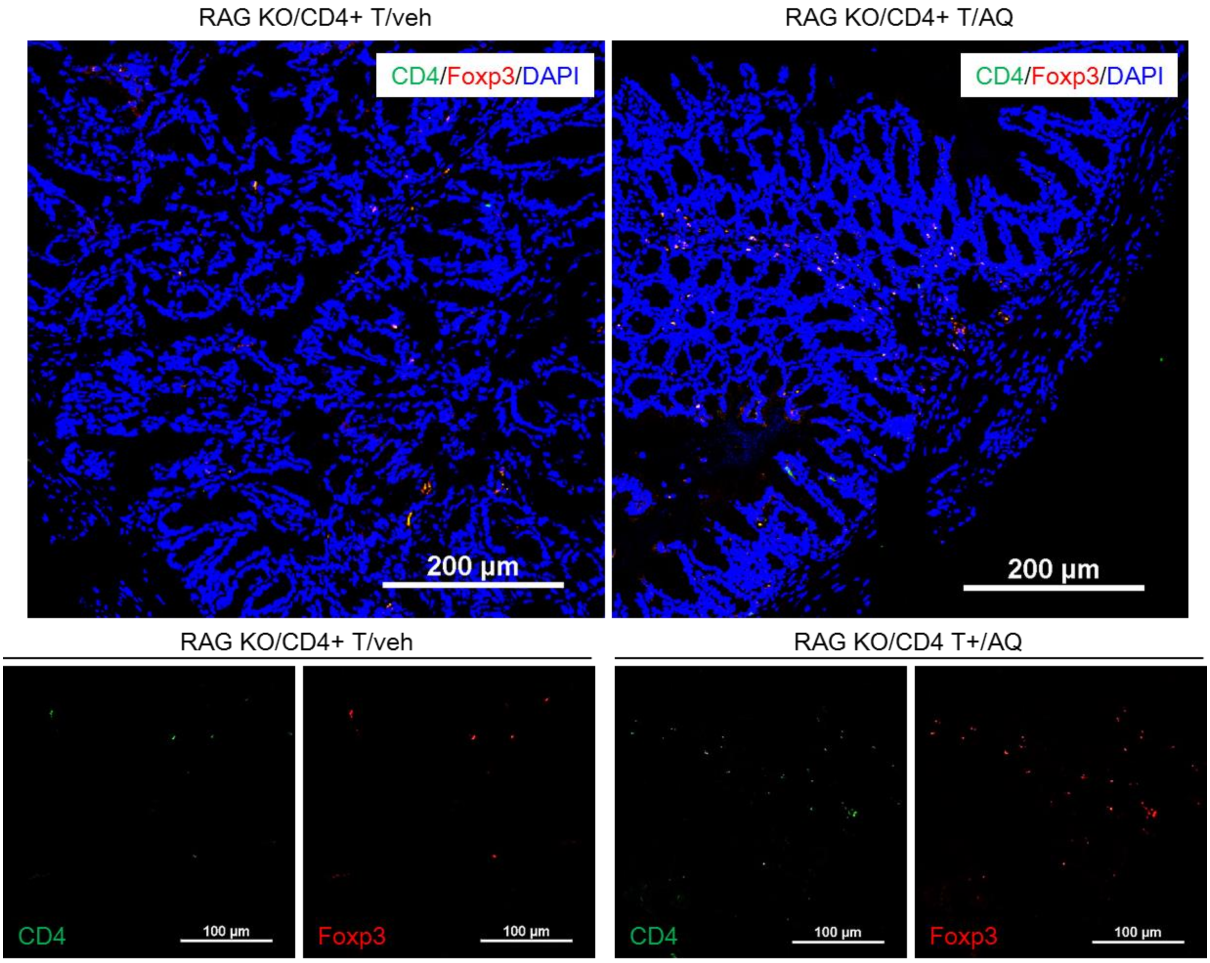

Supplementary Fig. S7. Uncropped blots in Figure 7F

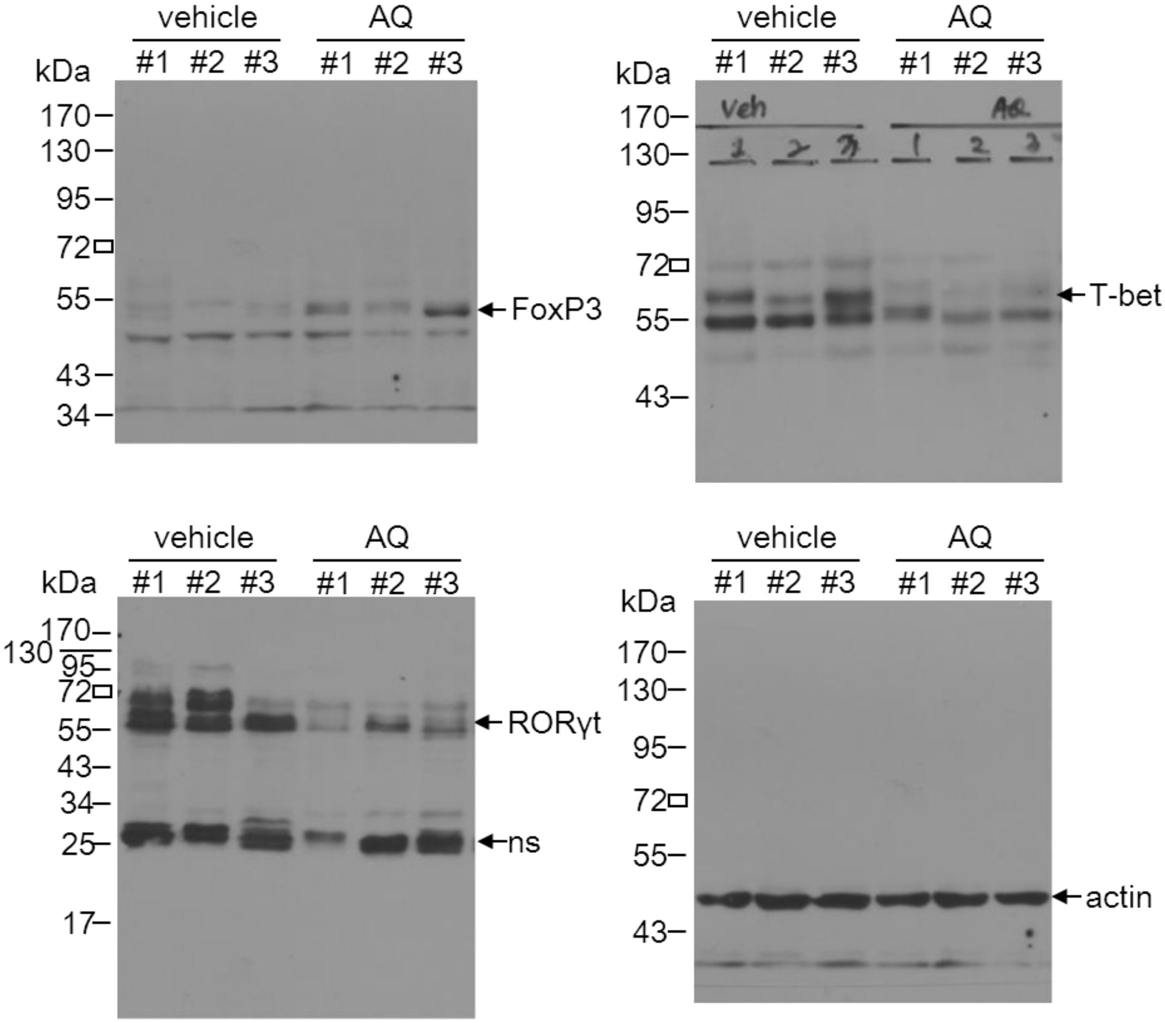

Supplement: Supplementary file 1 — Supplementary Figure [file 41598_2017_17073_MOESM1_ESM.pdf]
